# Annual Report of the Managers of the (Kentucky) Lunatic Asylum

**Published:** 1845-05

**Authors:** 


4. The present report is the first that has been made by Dr. Allen, since his appointment to the Superintendence of the Kentucky Asylum, and we welcome it as an indication of the success which may reasonably be expected to reward his labours in their administration. This Institution, if we may judge from its previous reports and some statistics in the one before us, has hitherto been in bad hands; its friends may therefore congratulate themselves on having secured the services of a gentleman who seems in every way qualified to discharge the responsible duties of the station.

On the 1st March, 1844, there were in the Asylum,—

| | Men. | Women. | Total. |
|---|---|---|---|
| | 92 | 71 | 163 |
| Admitted since, | 46 | 27 | 73 |
| Under care during the year, | 138 | 98 | 236 |
| Discharged " " | 36 | 17 | 53 |
| Remaining, | 102 | 81 | 183 |

Of the 53 cases discharged 34 were restored and 8 died.

The following extract will give an idea of the state of things that has hitherto existed, in comparison with the present.

"Table No. 3.—Shows the number of deaths each month and year, since 1824 to December 31, 1844, inclusive, which is 416, or about 1 in 2¼—an unusually large proportion. This great mortality seems to have attended this establisnment up to the present year, and during this year not a very small proportion have died. For three years past, 1840, 1841, 1842, and 1843, including January and February, 1844, there have occurred 105 deaths, about 1 in 4. During the present year, (since 1st March last) we have had 8 deaths out of 237, about 1 in 29½."

The greater part of the report is occupied with an account of the present condition of the Asylum, its defects and wants; earnestly calling the attention of the Legislature to the importance of making it, what every Institution for the insane ought to be, an establishment for curing the disease, and not a receptacle merely for incurable cases. J. H. W.

